# Advanced Targeted, Cell and Gene-Therapy Approaches for Pediatric Hematological Malignancies: Results and Future Perspectives

**DOI:** 10.3389/fonc.2013.00106

**Published:** 2013-04-30

**Authors:** Chiara F. Magnani, Sarah Tettamanti, Francesca Maltese, Nice Turazzi, Andrea Biondi, Ettore Biagi

**Affiliations:** ^1^Department of Pediatrics, Centro di Ricerca Matilde Tettamanti, San Gerardo Hospital, University of Milano-BicoccaMonza, Italy

**Keywords:** advanced therapy, pediatric leukemia, novel drugs, adoptive immunotherapy, chimeric antigen receptor, gene therapy

## Abstract

Despite the survival of pediatric patients affected by hematological malignancies being improved in the last 20 years by chemotherapy and hematopoietic stem cell transplantation, a significant amount of patients still relapses. Treatment intensification is limited by toxic side effects and is constrained by the plateau of efficacy, while the pipeline of new chemotherapeutic drugs is running short. Therefore, novel therapeutic strategies are essential and researchers around the world are testing in clinical trials immune and gene-therapy approaches as second-line treatments. The aim of this review is to give a glance at these novel promising strategies of advanced medicine in the field of pediatric leukemias. Results from clinical protocols using new targeted “smart” drugs, immunotherapy, and gene therapy are summarized, and important considerations regarding the combination of these novel approaches with standard treatments to promote safe and long-term cure are discussed.

## Introduction

Current treatments of childhood hematological malignancies are based on standardized regimens with poly-chemotherapeutic drugs, developed over the last 40 years. Significant progress has been achieved in this field, particularly as a result of the combination with hematopoietic stem cell transplantation (HSCT) which led to an increase of more than 80% in survival rate of acute lymphoblastic leukemia (ALL) and to a greater than 60% remission of the less common acute myeloid leukemia (AML) (Wayne et al., 2008). Changes in the timing scheme together with dose scaling, combination of different chemotherapeutic drugs, and modifications in their formulations resulted in multi-agent-chemotherapy protocols, which allowed better disease management and increased survival prognosis (Lee-Sherick et al., 2010). Immunotherapy with HSCT in high-risk patients has decreased the risk of relapse due to its strong graft-versus-leukemia (GVL) effects, even if it often correlates with a higher incidence of treatment-related mortality (Leung et al., 2011).

Along with cytotoxic treatments, high standards of supportive care, particularly antimicrobial prophylaxis (Unguru, 2011), have highly improved the quality of the current therapeutic approaches. Further progresses have been reached also thanks to the translation of recent findings from bench to bedside, paving the way for the development of novel randomized clinical trials involving patients with more aggressive diseases. Furthermore, patients diagnosed with leukemia are treated with a step-by-step protocol based on the characteristics of the disease at onset and the minimal residual disease (MRD) that is detected after first-line drugs. Such a strategy of risk-oriented stratification is the first step toward the concept of personalized medicine and is currently leading to lower rate of relapse, particularly in ALL (Locatelli et al., 2012).

Despite the important efficacy of the current treatments, relapse still occurs and a significant number of patients falls back (Wayne et al., 2008). Escalation of the current treatments seems not to add any further advantage. In these cases, alternative treatments based on targeted agents and advanced protocols of gene and adoptive cell therapy (ACT) are strongly warranted.

In this review, we aim to summarize novel advanced drugs and treatments that are available or are considered promising strategies. In addition we provide proof of concept for future integration of several novel approaches and standard treatments in a context of “consolidative immunotherapy.”

## New Antimetabolites, Nucleoside Analogs, and “Smart” Drugs

Nowadays antimetabolites are considered one of the most effective category of drugs available for treating hematological malignancies. Clofarabine was synthesized as a next-generation purine nucleoside analog (Montgomery et al., 1992; Kantarjian et al., 2007). In 2004 this drug was approved by the FDA for the treatment of pediatric relapsed or refractory ALL patients. Phase I and II trials with clofarabine showed an overall response rate of 30% as single agent for refractory pediatric ALL and AML (Jeha et al., 2004, 2006). Combination with cyclophosphamide/etoposide, evaluated in phase I (Hijiya et al., 2009), phase II (Locatelli et al., 2009; Hijiya et al., 2011), and phase III trials (NCT01406756)^1^, revealed encouraging overall response rates, near 50% for ALL and 100% for five AML patients, despite significant treatment-related adverse effects, such as infections, neutropenia, and hepatotoxicity. Decitabine, a DNA methyltransferases inhibitor (Pinto et al., 1984; Schafer et al., 2010), has been used as epigenetic priming in combination with chemotherapy in kids with AML (NCT01177540; NCT00943553). Preliminary data demonstrated that the treatment is well tolerated; however, clinical responses were similar to the control arm of the study (Gore et al., 2012). Novel and advanced treatments against hematological malignancies focus on targeted therapy, characterized by selection of specific molecular targets. Such drugs are considered “smart” since they selectively target cancer signaling pathways or expression of genes specifically overexpressed in cancerous and not healthy cells (Villanueva, 2012). Among them, tyrosine kinase inhibitors (TKI), which act as signal transduction inhibitors, target enzymes overexpressed by malignant tumors involved in uncontrolled cell proliferation, inhibition of apoptosis, and cell adhesion (Hunter, 1998; Arora and Scholar, 2005). Early success of the first targeted agent Imatinib mesylate in CML and Ph^+^ ALL treatment (O’Brien et al., 2003; Wassmann et al., 2004) demonstrated the efficacy of inhibiting BCR/ABL1 (p210), the constitutive active kinase protein produced by the abnormal fusion of the two genes. These results were confirmed also in pediatric patients with Ph^+^ ALL, improving 3-year event-free survival (EFS) from 35% of the historical controls to 80% with no increased toxicity (Schultz et al., 2009). However, the presence of mechanisms of Imatinib resistance and transient responses (Walz et al., 2006) encouraged the development of second generation TKI, such as Dasatinib (Olivieri and Manzione, 2007), Nilotinib (Weisberg et al., 2005), and Bosutinib (Redaelli et al., 2009). A pediatric phase I trial on Ph^+^ CML and ALL patients carried out by the Children’s Oncology Group (COG) revealed that the majority of children with CML achieved a clinical response after oral administration of Dasatinib (Aplenc et al., 2011). Two more phase II and one phase III pediatric clinical trials are currently ongoing with the aim to evaluate whether Dasatinib is safe and effective in the treatment of Ph^+^ ALL and CML, alone or in combination with standard chemotherapy (NCT00777036; NCT01460160; NCT00720109). In this context, continuous dose Dasatinib has been shown to be safe and feasible in combination with intensive chemotherapy in pediatric Ph^+^ ALL (Slayton et al., 2012), demonstrating that early introduction of TKI on day 15 of induction and substitution of Dasatinib for Imatinib led to improved induction remission rates from 89 to 98%, and induction negative MRD rates from 25 to 59%. Certainly, Dasatinib represents one of the most promising drugs in the context of small smart molecules for refractory Ph^+^ ALL.

Another important molecule is represented by FMS-like tyrosine kinase 3 (FLT3), in which point mutations and internal tandem duplications lead to the encoding of a constitutive active kinase, both in childhood ALL and AML, which is associated with poor prognosis (Levis and Small, 2003). Promising FLT3 inhibitors currently tested for childhood malignancies are Lestaurtinib (Levis et al., 2002), Sorafenib (Rubnitz, 2012), and Midostaurin (Fabbro et al., 2000). Sorafenib has been recently evaluated in combination with clofarabine and cytarabine, showing response in pediatric relapsed/refractory AML (Inaba et al., 2011). Trials evaluating oral Lestaurtinib (phase III) in infant mixed lineage leukemia-rearranged (MLL-R) ALL, Sorafenib (phase III) in patients with AML, and Midostaurin (phase I/II) in relapsed pediatric leukemia are currently recruiting patients (NCT00557193; NCT01371981; NCT00866281).

## Cancer Immunotherapy: Monoclonal Antibodies and Cell Therapy

Over the last decades, significant progress in understanding the complex involvement of the immune system in tumor surveillance has been made. This has led to novel approaches that exploit both the humoral and cell-mediated arms of adaptive immunity, such as monoclonal antibodies (mAbs), cancer vaccines, and ACT. The ultimate goal of immunotherapy is to decrease toxicity against normal tissues by eliciting specific responses against tumor associated-antigens (TAA) and to bypass the tumor immune escape/tolerance mechanisms (Dougan and Dranoff, 2009).

Rituximab (anti-CD20) (Dworzak et al., 2008) is currently applied for the treatment of CD20^+^ B-cell lymphomas; several *mAbs* targeting different TAAs have been proposed for pediatric patients in association with chemotherapy, such as anti-CD52 [Alemtuzumab (Law et al., 2012)] and CD22 [Epratuzumab (Scott et al., 2012)]. Rituximab has demonstrated to be active as single agent in pediatric B-NHL with 41.4% response rate (Meinhardt et al., 2010) and combined with chemotherapy in CD20^+^ NHL and leukemia with 60% response rate (Griffin et al., 2009). Alemtuzumab was evaluated as single agent in children with relapsed ALL, showing limited response with only 1 patient in complete remission out of 13-tested (Angiolillo et al., 2009). Thus, given the limitations of using mAbs as single agents in rapidly proliferative diseases, mAbs conjugated with cytotoxic agents, including *antibody-drug conjugates (ADCs)*, *immunotoxins*, and *radioimmunoconjugates* (FitzGerald et al., 2011; Sharkey and Goldenberg, 2011), have been developed and tested in clinical trials, showing promising results in adults (Mackall, 2011; Orentas et al., 2012). The anti-CD22 immunotoxin, Moxetumomab pasudotox, achieved 29% response rate (Wayne et al., 2011), whereas the anti-CD22 calicheamicin conjugate, Inotuzumab ozogamicin, showed 57% response rate (Kantarjian et al., 2012) in relapsed/refractory ALL pediatric patients. The ADC Brentuximab vedotin in Hodgkin’s lymphoma has been approved for commercial use after it demonstrated significant activity in adults (Younes et al., 2010) and it is currently evaluated in several pediatric clinical trials. Notably, the novel bi-specific T-cell engager (BiTE) represents, among all, one of the most promising approaches. This antibody simultaneously cross-links the CD19^+^ target and the CD3^+^ T cells, recruiting the effector cells to the tumor. A multicenter study involving Northern American and European institutions is ongoing (May et al., 2012). Table 1 summarizes the use of mAbs, ADCs, immunotoxins, and radioimmunoconjugates in pediatric clinical trials.

**Table 1:** **The list represents a selection of the main trials for each single agent for novel approaches in pediatric hematological malignancies in the field of mAbs and derivates**.

Target	Malignancies	mAbs, ADCs, immunotoxins, radioimmunoconjugates	Published results	Location	Trial number and references
CD19	Advanced relapsed/refractory ALL or CLL	Yttrium Y 90 anti-CD19 monoclonal antibody BU12/111In-BU-12	Terminated due to slow accrual	Masonic Cancer Center, University of Minnesota	NCT00643240 Phase I

CD19^+^ CD3	Relapsed/refractory precursor B-cell ALL	Blinatumomab (BiTE = bi-specific antibodies)	Currently recruiting participants	Amgen Research (Munich) GmbH, Multicentric study	NCT01471782 Phase I/II

CD20	Recurrent/refractory NHL and ALL	Rituximab (Rituxan^®^; MabThera) associated to chemotherapy	Completed, 20 patients, CR/PR 12/20 (60%)	National Cancer Institute (NCI)	NCT00058461 Phase II (Griffin et al., 2009)
	B-cell ALL and NHL	Rituximab	87 patients, 41.4% ORR	Kinderklinik, Aachen, Multicentric study	NCT00324779 Phase II (Meinhardt et al., 2010)
	Relapsed/refractory precursor B-cell ALL and lymphoma	Rituximab associated to chemotherapy and haploidentical NK cell infusion	Currently recruiting participants	St. Jude Children’s Research Hospital	NCT01700946 Phase II
	Relapsed ALL	Rituximab associated to chemotherapy	The study has been terminated	Emory University Multicentric study	NCT01230788 Phase I
	Refractory leukemia and lymphoid malignancies involving the central nervous system	Intrathecal rituximab	Currently recruiting participants	M.D. Anderson Cancer Center	NCT01596127 Phase I/II
	B-cell ALL and NHL	Rituximab associated to chemotherapy	Currently recruiting participants	Institut Gustave Roussy	NCT01516580 Phase III

CD22	ALL, NHL	Moxetumomab pasudotox (HA22; CAT80-15)	Currently recruiting: 21 patients treated, 24% CR, 1% PR	National Cancer Institute (NCI) Multicentric study	NCT00659425 Phase I (Wayne et al., 2011)
	Relapsed/refractory ALL	Inotuzumab ozogamicin (CMC-544) with or without rituximab	Ongoing, 49 patients treated (range 6–80 years), 57% ORR	M.D. Anderson Cancer Center	NCT01134575 Phase I (Kantarjian et al., 2012)
	Relapsed ALL	Epratuzumab associated to chemotherapy	Ongoing, but not recruiting participants	National Cancer Institute (NCI) Multicentric study	NCT00098839 Phase II

CD30	Anaplastic large-cell lymphoma	mAbs SGN-30 associated to chemotherapy	This study has been completed	National Cancer Institute (NCI)	NCT00354107 Phase I/II
	HL anaplastic large-cell lymphoma	Brentuximab vedotin (SGN-35)	Currently recruiting participants	Millennium Pharmaceuticals, Inc. Multicentric study	NCT01492088 Phase I/II
	Hodgkin lymphoma, large-cell, anaplastic lymphoma, non-hodgkin		Approved for sale to the public	Seattle Genetics, Inc. Multicentric study	NCT01196208
	ALL, AML, CLL, MM, solid tumors		Currently recruiting participants	Seattle Genetics, Inc. Multicentric study	NCT01461538 Phase II

CD33	Newly diagnosed AML	Gemtuzumab ozogamicin	Ongoing	National Cancer Institute (NCI) Multicentric study	NCT00372593 Phase III

CD52	Recurrent childhood acute lymphoblastic leukemia	Alemtuzumab (Campath-1H) associated to chemotherapy	Limited response: 8% ORR	Children’s Oncology Group, Arcadia, CA, USA	NCT00089349 Phase II (Angiolillo et al., 2009)

*CR, complete response; PR, partial response; ORR, overall response rate*.

The promise of eliciting specific memory response by active immunotherapy using *cancer vaccines* has guided the development of different approaches, including vaccines composed of tumor-specific peptides, dendritic cells (DC) pulsed with peptides, and whole tumor cells (Rousseau and Brenner, 2005; Wayne et al., 2010). The results of the first three treated pediatric cases in the phase II trial using WT1 peptide vaccination plus HSCT showed improved GVL effect and increased percentages of WT1-specific cytotoxic T-lymphocytes (CTLs) and no treatment-related adverse events. Nevertheless, the treatment failed to achieve effectiveness in patients with active disease (Hashii et al., 2010, 2012). A trial with DCs pulsed with WT1 is ongoing at NIH (NCT00923910) and adult as well as pediatric patients are being recruited. Unfortunately, despite highly promising, antigen-specific vaccination strategies have shown limited efficacy thus far, especially as single therapies.

The approach of *ACT* using Tumor Infiltrating Lymphocytes (TIL) (Rosenberg et al., 1986), allogeneic HSCT, or Donor Lymphocyte Infusions (DLI) (Weiden et al., 1981) comprises the powerful features of adaptive immunity and provides GVL effect. In the perspective of defining the best cell population to be infused in patients, since DLI may cause graft-versus-host disease (GvHD), different subsets of *CTLs* (Montagna et al., 2008), and *natural-killer (NK)* (Locatelli et al., 2013) cells have been isolated and studied to define their specificity, toxicity, and *in vivo* persistence over time. Haploidentical NK cell infusions after an immunosuppressive regimen were well tolerated and resulted in successful engraftment in a pilot study with children affected by AML (Rubnitz et al., 2010). Among novel effector cells, *Cytokine-Induced Killer cells* (CIK), a peculiar natural-killer like population with a basal anti-tumor activity, are under investigation in clinical trials^2^. In 2007 our group demonstrated that the adoptive transfer of allogeneic CIK cells is feasible under clinical grade conditions and well tolerated (Introna et al., 2007). Similar results were obtained for haploidentical CIK cell infusions in pediatric patients (Rettinger et al., 2013). An open-labeled, multicenter phase II study involving both adult and pediatric patients has been recently concluded with promising results at the highest CIK cell dose infused, with limited toxicity (Introna et al., 2011). Of particular relevance is the recent identification of the *T-stem cell memory* (T_SCM_) subset with enhanced proliferative and anti-tumor activity. T_SCM_ have stem cell-like properties of self-renewal capacity and multipotency (Gattinoni et al., 2011), they can be derived and expanded *in vitro*, offering a promising platform of cellular production for future translation in clinic (Cieri et al., 2013).

## Cancer Immunotherapy with Gene Transfer: TCR and CAR

Unmanipulated T- or NK-cells have proven to have several limitations after infusion both in terms of limited activity and poor long-term survival. Therefore, in the last years, there has been considerable interest in the development of fine strategies of gene transfer to genetically manipulate immune cells and improve their anti-tumor immune responses *in vivo*. In this context, *artificial T-cell receptors* (TCR) and *Chimeric Antigen Receptors* (CARs) have been generated to redirect effector immune cells specifically against TAAs (Gross et al., 1989; Clay et al., 1999).

Transfer of engineered T cells with artificial high-affinity TCR derived from α and β chains isolated from patients has been used in successful clinical trials by Rosenberg and collaborators, targeting MART1 (Morgan et al., 2006) or NY-ESO-1 (Robbins et al., 2011) for the treatment of melanoma and synovial cell sarcoma. However, since this approach is limited by tumor escape mechanisms, scientific efforts were taken for optimizing the functionality of the artificial TCR. Notably, an emerging technical advance concerning this strategy has been recently reported: TCR editing was optimized by zinc-finger nucleases that eliminate the risk of TCR mispairing with endogenous α and β chains, which would otherwise cause an unpredictable and thus not safe specificity (Provasi et al., 2012).

Chimeric antigen receptors are chimeric TCR that are artificially constituted by an antigen-recognizing extracellular domain derived from an antibody, linked to a T-cell triggering domain and are introduced in effector T cells to redirect their activity toward TAA (Gross et al., 1989). In contrast to artificial TCR strategy, target recognition by CAR molecules is non-HLA restricted and independent of antigen processing, bypassing HLA-molecule down-regulation. Although the first clinical trials with CAR demonstrated the feasibility of this approach to target hematological malignancies in terms of safety and tolerability, they also highlighted the need to improve the *in vivo* persistence of the transferred T cells (Till et al., 2008). Therefore, second and third generation CARs have been developed, by addition of one or two co-stimulatory molecules (Lee et al., 2012a).

To enhance the survival and activity of modified T cells, expression of CAR or TCR was performed in Epstein Barr Virus (EBV)-specific CTLs. Due to their dual specificity longer cell survival and enhanced tumor regression were achieved, as a result of the continuous engagement of the native (EBV-specific) TCR on professional APCs (Pule et al., 2008). However, these approaches still need to be implemented in terms of efficacy and safety (Park et al., 2011) since serious adverse events due to “on-target but off-organ” toxicity occurred in two clinical trials with CARs targeting HER2 and CD19 (Heslop, 2010). The introduction of suicide genes, such as inducible Casp9, within the engineered T cells could be an additional back-up control in case of adverse effects (Di Stasi et al., 2011) and a clinical trial is ongoing to treat patients developing GVHD after HSCT (NCT00710892).

Nowadays we are witnessing a new era of ACT, when recent successes in clinical trials with CAR reinforced the potential therapeutic benefit of this approach. Several clinical trials are ongoing in pediatric patients, starting from evidence of tumor regression in four out of eight patients belonging to studies with EBV-specific CTLs expressing GD2-specific CARs for the treatment of neuroblastoma (Pule et al., 2008) (NCT01460901). Table 2 summarizes the main pediatric clinical trials with CAR for the treatment of hematological malignancies that are actually ongoing. A trial with CD19-targeting CAR EBV-specific T cells showed important preliminary results suggesting the feasibility of this approach without infusion-related toxicity (Curran et al., 2012). Notably, Grupp and collaborators recently reported induction of remission followed by B-cell aplasia and Cytokine Release Syndrome (CRS) in two pediatric patients with relapsed, refractory pre-B-cell ALL treated with CD19-specific CAR T cells. In one patient CRS was controlled by administration of the IL-6 antagonist Tocilizumab and remission is still ongoing whereas the other patient relapsed with the emergence of CD19-negative blasts (Grupp et al., 2013). Our group has been involved in the “CHILDHOPE” program, a translational research project focused on the treatment of childhood ALL, lymphoma, and AML with CD19- and CD33-specific CARs, respectively^3^. Furthermore, for the treatment of AML, we are currently investigating the targeting of CD123 antigen in order to improve the specificity and safety of the CAR approach (Tettamanti et al., 2013).

**Table 2:** **Ongoing pediatric clinical trials using CARs**.

Target	Malignancies	Intervention	Results	Location	Trial number and references
CD19	ALL	Anti-CD 19 CAR donor EBV-CTL post-HSCT with EBV-CTL vaccine	Currently recruiting participants	University College of London Multicentric study	NCT01195480 Phase I/II

CD19	ALL	Anti-CD 19 CAR donor EBV-CTL post-HSCT	Currently recruiting participants, three patients treated without GvHD after infusion	Memorial Sloan–Kettering Cancer Center	NCT01430390 Phase I (Curran et al., 2012)

CD19	B-cell malignancy: ALL, B-cell lymphoma, leukemia large-cell lymphoma, NHL	Anti-CD 19 CAR autologous PBL	Currently recruiting participants, one CR pediatric ALL patient after a mild CRS	National Cancer Institute	NCT01593696 Phase I (Lee et al., 2012b)

CD19	B-cell leukemia, B-cell lymphoma	Pedi CART-19: anti-CD 19 CAR second generation (4-1BB) autologous PBL	Currently recruiting participants, two CR pediatric ALL patients	Children’s Hospital of Philadelphia/University of Pennsylvania	NCT01626495 (Grupp et al., 2013)

CD19	B-cell leukemia	Anti-CD 19 CAR first generation autologous PBL	Currently recruiting participants	Seattle Children’s Hospital	NCT01683279 Phase I

## Future Perspectives

The recent efficacy of targeted therapies has changed our perspective of leukemia treatment. New-generation small molecules, such as Dasatinib and lately designed mAbs, such as Inotuzumab or BiTe represent major progress toward cure, but their success is partially eclipsed by the drawbacks of resistance or transient response to therapy (Walz et al., 2006). Advanced protocols of gene and ACT may help to overcome these limitations. Actually, these approaches should promote long-term efficacy, maintaining specificity with reduced toxicity. Moreover, improvements in novel technologies of drug delivery, such as nanoparticles (Acharya and Sahoo, 2011), or easier and more efficient methods of gene transfer, such as transposons (Izsvak et al., 2009), should further ameliorate the range of applications of these novel therapies. Indeed, a new phase I clinical trial with genetically modified human T cells expressing anti-CD19. CAR using the Sleeping Beauty transposon system is actually ongoing in patients with high-risk B-lymphoid malignancies (Kebriaei et al., 2012).

The scenario that we expect to see in the next future is the development of advanced protocols in the context of “consolidative therapy.” Immunotherapy by gene-redirected immune cells will provide the potential of controlling MRD in patients following initial chemotherapy or HSCT, behaving as a “long-lasting living” drugs, contrarily to standard chemotherapy agents or mAbs. Phase I and II clinical trials are currently combining chemotherapy and HSCT with targeted therapy or immunotherapy for patients who failed standard treatments. These studies will assess safety, efficacy, and feasibility in applying such combined approaches. The next step will be the definition of the best timing schedule and dosing regimen in patients that will truly benefit from these immuno-gene-therapy approaches. Over the next decade, clinicians and scientists will have the unique chance to witness the effects of advanced treatments in pediatric patients affected by hematological malignancies.

## Conflict of Interest Statement

The authors declare that the research was conducted in the absence of any commercial or financial relationships that could be construed as a potential conflict of interest.

## Footnotes

^1^http://www.clinicaltrials.gov

^2^http://www.cik-info.org/index.php?kat = ircc—international-registry-on-cik-cells

^3^www.childhope.eu
